# Comparative transcriptome analysis of the rice leaf folder (*Cnaphalocrocis medinalis*) to heat acclimation

**DOI:** 10.1186/s12864-020-06867-6

**Published:** 2020-06-30

**Authors:** Peng-Qi Quan, Ming-Zhu Li, Gao-Rong Wang, Ling-Ling Gu, Xiang-Dong Liu

**Affiliations:** Department of Entomology, Nanjing Agricultural University, Nanjing, 210095 China

**Keywords:** Heat acclimation, Heat stress response, Metabolism, Rice leaf folder, Sense, Transcriptome

## Abstract

**Background:**

The rice leaf folder *Cnaphalocrocis medinalis* Güenée is a serious insect pest of rice in Asia. This pest occurs in summer, and it is sensitive to high temperature. However, the larvae exhibit heat acclimation/adaptation. To understand the underlying mechanisms, we established a heat-acclimated strain via multigenerational selection at 39 °C. After heat shock at 41 °C for 1 h, the transcriptomes of the heat-acclimated (S-39) and unacclimated (S-27) larvae were sequenced, using the unacclimated larvae without exposure to 41 °C as the control.

**Results:**

Five generations of selection at 39 °C led larvae to acclimate to this heat stress. Exposure to 41 °C induced 1160 differentially expressed genes (DEGs) between the heat-acclimated and unacclimated larvae. Both the heat-acclimated and unacclimated larvae responded to heat stress via upregulating genes related to sensory organ development and structural constituent of eye lens, whereas the unacclimated larvae also upregulated genes related to structural constituent of cuticle. Compared to unacclimated larvae, heat-acclimated larvae downregulated oxidoreductase activity-related genes when encountering heat shock. Both the acclimated and unacclimated larvae adjusted the longevity regulating, protein processing in endoplasmic reticulum, antigen processing and presentation, MAPK and estrogen signaling pathway to responsed to heat stress. Additionally, the unacclimated larvae also adjusted the spliceosome pathway, whereas the heat-acclimated larvae adjusted the biosynthesis of unsaturated fatty acids pathway when encountering heat stress. Although the heat-acclimated and unacclimated larvae upregulated expression of heat shock protein genes under heat stress including *HSP70*, *HSP27* and *CRYAB*, their biosynthesis, metabolism and detoxification-related genes expressed differentially.

**Conclusions:**

The rice leaf folder larvae could acclimate to a high temperature via multigenerational heat selection. The heat-acclimated larvae induced more DEGs to response to heat shock than the unacclimated larvae. The changes in transcript level of genes were related to heat acclimation of larvae, especially these genes in sensory organ development, structural constituent of eye lens, and oxidoreductase activity. The DEGs between heat-acclimated and unacclimated larvae after heat shock were enriched in the biosynthesis and metabolism pathways. These results are helpful to understand the molecular mechanism underlying heat acclimation of insects.

## Background

Mean land surface air temperature has increased by 1.53 °C from 1850 to 1900 to 2006–2015 [1]. Insects are prone to heat-related injuries [2–4]. The increased temperature significantly affects performance of insect populations [5, 6]. Fitness-related traits of caterpillars *Lobesia botrana* are sensitive to increasing temperature [6]. Temperature, to a great extent, determines the development, survival, reproduction and behaviour of insects [7–9]. For example, the development time of *Phthorimaea operculell* decreased whereas survival rate increased, as temperature increased from 17.5 to 27.5 °C, but the development stopped at 35 °C [9]. The longevity of adult *Diaphorina citri* significantly decreased at 41 °C, and approximate 20% adults survived for only 2 h at this high temperature [8]. The high temperature of 41.8 °C inhibited nymphal development of the brown planthopper *Nilaparvata lugens*, and the exposure to 42.5 °C in adult stage resulted in a lower fecundity and extended developmental duration of eggs [7]. Temperature also affects insect behaviours, such as feeding [3], flight and walking [10], host choice, settling and folding leaf behaviours [11]. Changes in temperature also result in deficiency or abnormality of insects in respiration, nervous, metabolism, and endocrine systems [12–14]. The ventilatory rhythm frequency of the locust *Locusta migratoria* increases with the increase of temperature [12]. The resting metabolic rates of the wood tiger moths *Arctia plantaginis* are significantly higher when larvae reared at 25 °C than that at 16 °C [13]. Temperature affects invertebrate hormone system, and the increased temperature induces expression of endocrine signaling genes of chironomids *Chironomus riparius* [14].

Although temperature affects insect performance, insects have a certain ability to adapt to an unsuitable thermal condition. The grain aphid *Sitobion avenae* [15], green peach aphid *Myzus persicae* [16], and silk worm *Bombyx mori* [17] increase adaptability or resistance to an extreme temperature when they have experienced another temperature approaching to the extreme one. Under high temperature conditions, evaporative cooling improves insect thermotolerance [18]. A general cellular response of insects to high temperature is the inductive heat shock proteins (Hsps) which protect insects from heat injuries [19, 20]. For example, an upregulation of gene expression of the *Hsp40* was found in thermotolerant lines of *Drosophila melanogaster* when they subjected to a mild heat shock [21]. Survival rates and *Hsp70* gene expression levels of two *Drosophila buzzatii* populations collected from the high- and low-temperature environments are different when they are exposed to 39 °C, which shows genetic differences in thermal tolerance between populations [22]. However, the molecualr mechanisms underlying physiological and molecular responses or acclimation to heat stress are still largely unknown.

Sensation-related genes involve the responses of insects to heat stress [23, 24]. In *Drosophila*, the gustatory receptor GR28B(D) drives the rapid response of flies exposed to a steep warmth gradient, and GR28B(D) misexpression confers thermosensitivity upon diverse cell types [23]. Ants *Temnothorax* can adjust their cuticular hydrocarbon profile to acclimate to different temperatures [25]. Approximately 58% of the odorant binding proteins *obps* genes in the antenna of *Drosophila* exhibit a change in expression after heat treatment [24]. Expression levels of sensation-related genes may contribute to heat acclimation of insects.

The rice leaf folder *Cnaphalocrocis medinalis* (Lepidoptera: Pyralidae) is an important pest of rice and other gramineous crops in Asia, often causing serious losses [26]. This pest is sensitive to temperature changes [27–29]. The upper and lower threshold temperatures of this pest are 36.4 °C and 11.2 °C, respectively [30]. Eggs of the rice leaf folder can not hatch at 37 °C [31]. Survival rate of the first-instar larvae is more than 60% when exposed to 39 °C, but it is only 20% at 41 °C [28]. Moreover, high temperature affects host preference and shelter-building behaviour of the rice leaf folder larvae [11, 32, 33]. The longevity and copulation frequency of adults, and hatchability of eggs are significantly reduced when adults exposed to 39 °C or 40 °C [27]. Although the rice leaf folders are susceptible to heat stress, the population outbreaks still occur frequently under global warming [34, 35]. A previous study illustrated that the rice leaf folder larvae could increase their heat tolerance via heat selection, and heat shock protein genes were upregulated in the selected larvae [36]. This result implies that the rice leaf folders have potential to acclimate or adapt to heat stress. However, the gene expression profiles of larvae to respond and acclimate to heat stress are still unknown. Therefore, in this study, we successively selected the 3rd instar larvae at 39 °C for several generations, and a heat-acclimated strain was generated which showed the similar survival rate under the 39 °C treatment as the control at 27 °C. Then, we sequenced and analyzed the transcriptome of the 3rd instar larvae collected from the heat-acclimated strain (S-39) and the unacclimated strain (S-27) after exposure to 41 °C for 1 h, and the larvae from the unacclimated strain maintained at 27 °C was the control (CK). The differentially expressed genes (DEGs) in the S-39 vs CK, S-27 vs CK, and S-39 vs S-27, and GO and KEGG enrichment analyses were performed. The object of this experiment was to address the genes and pathways involving in the heat response and acclimation of the rice leaf folder larvae, which would highlight the molecular mechanism underlying heat acclimation.

## Results

### Heat acclimation of larvae

**Fig. 1:**
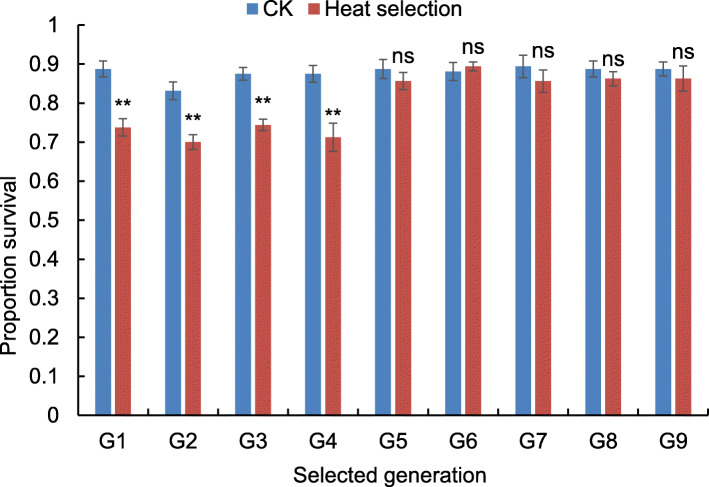
Survival rates of the 3rd instar larvae after 3 days of heat treatment at 39 °C for 3 h per day in each generation. ** means significant difference between heat treatment and control at *P* = 0.01 level, and ns means no significant difference at the *P* = 0.05 level using the Mann-Whitney U test. The error bar represents the standard error (SE)

Heat exposure at 39 °C significantly affected survival of larvae (*F*_1, 8_ = 11.594, *P* = 0.009), and this effect was also dependent on the generation of heat selection (*F*_8, 126_ = 4.149, *P* < 0.001; Fig. 1). The survival rate of 3rd instar larvae after heat exposure to 39 °C was significantly lower than that of control at 27 °C during the first four generations of selection (G1: U = 3.00, *P* = 0.001; G2: U = 1.50, *P* < 0.001; G3: U = 1.00, *P* < 0.001; G4: U = 4.00, *P* = 0.002), but it became not-significantly different from control after five generations of selection (G5: U = 22.00, *P* = 0.328; Fig. 1). The result showed that the rice leaf folder larvae could acclimate to heat stress at 39 °C via multigenerational heat selection (Fig. 1).

### Quality of transcriptome assembly and functional annotation

A total of 489,393,984 raw reads were obtained from nine samples. After the raw reads were filtered, there were 146,899,648, 155,412,572, and 175,944,178 clean reads obtained in the CK, S-27, and S-39, respectively. The Q30 values ranged from 93.67 to 96.63%, and the base-position error rate of sequencing was 0.02% or 0.03%. The GC content ranged from 50.28 to 53.48% (Table S1). Transcriptome assembly generated 191,974 unigenes and 289,127 transcripts based on all the nine samples. The minimal length of unigenes and transcripts was 201 bp, and mean length was 791 bp and 1040 bp for the transcript and unigene, respectively (Table S2). The lengths of 53,857 unigenes (28.05%) and 57,943 transcripts (20.04%) were 501–1000 bp (Fig. S1). The comprehensive genetic function annotation showed that 191,974 unigenes were aligned with seven public databases (Table S3). 41.43% (79,541) unigenes were annotated in NR database, and 31.08% (59,675) annotated in GO database. 53.93% (103,535) unigenes were annotated in at least one out of seven databases, and 5% (9609) unigenes were annotated in all seven databases (Table S3). The BUSCO analysis showed a 93.56% of completeness of the transcriptome (Fig. S2). These data indicated that the quality of RNA-Seq data was high.

### Differentially expressed genes between heat-acclimated and unacclimated larvae after heat exposure

**Fig. 2:**
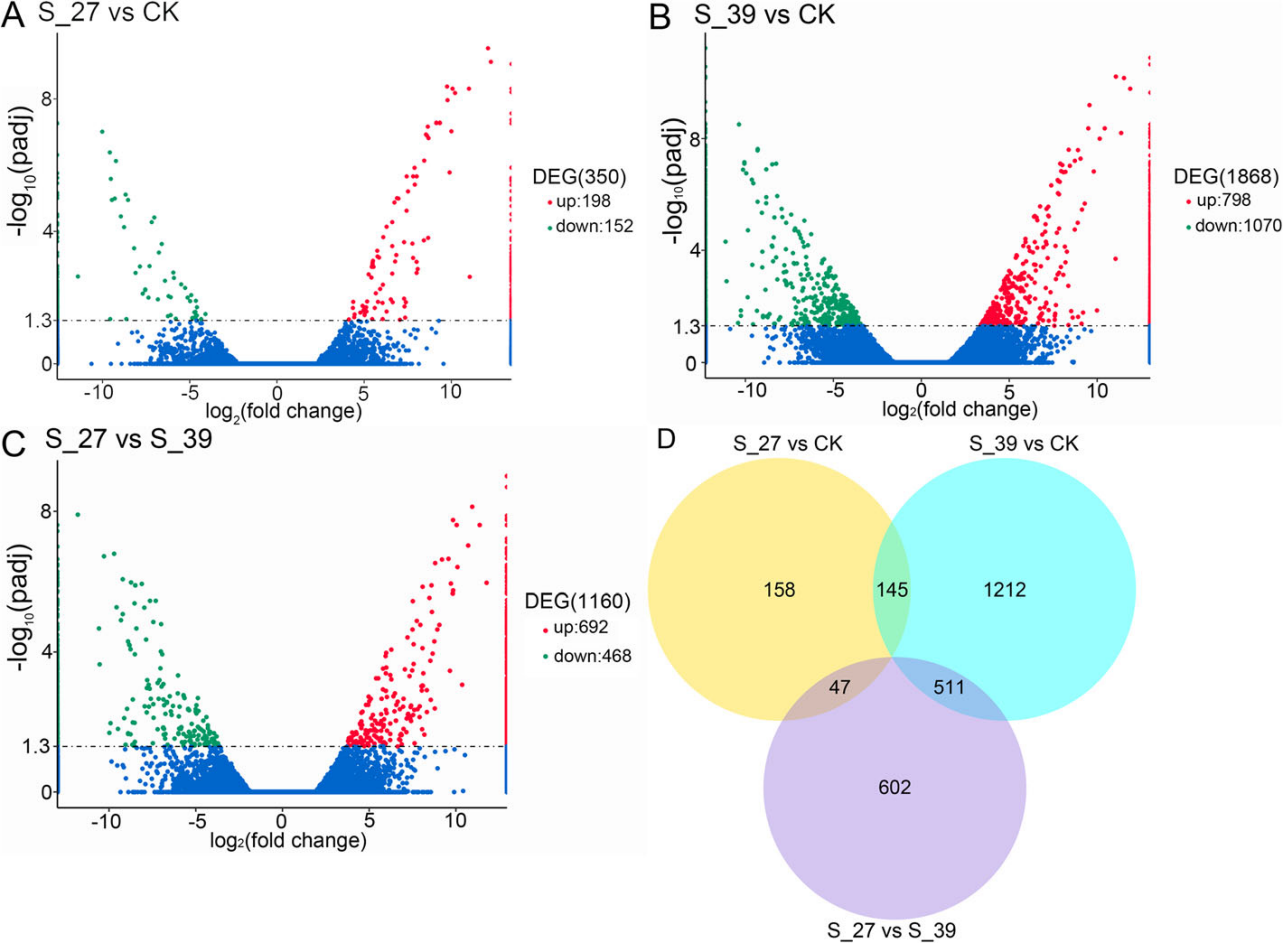
Volcano plot of differentially expressed genes between S-27 and CK (**a**), S-39 and CK (**b**), S-27 and S-39 (**c**), and the Venn diagram of these DEGs (**d**)

There were 350, 1868, and 1160 differentially expressed genes (DEGs) distributed in the comparison of S-27 vs CK (Fig. 2a), S-39 vs CK (Fig. 2b), and S-27 vs S-39 (Fig. 2c), respectively, based on a FDR corrected *p*-value of < 0.05. The heat-acclimated larvae (S-39) induced more DEGs to response to the heat exposure to 41 °C than the unacclimated larvae. A total of 2675 DEGs were found between S-39, S-27 and CK (Fig. 2d), The heat-acclimated larvae shared 145 DEGs with the unacclimated larvae after exposure to 41 °C, but they uniquely expressed 1723 DEGs other than the unacclimated larvae (Fig. 2d).

**Fig. 3:**
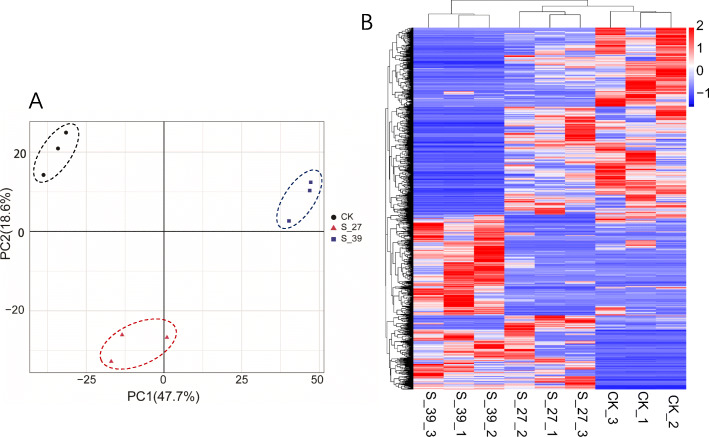
Principal component analysis (**a**) and hierarchical clustering (**b**) based on the FPKM of 2675 DEGs for the nine samples (CK-1, 2, 3; S-27-1, 2, 3 and S-39-1, 2, 3)

All three types of samples from the S-39, S-27, and CK could be distinguished using a principal component PC1 (47.7%) based on the FPKM of all DEGs. The values of PC2 (18.6%) could distinguish S-27 from S-39 and CK but could not distinguish S-39 from CK (Fig. 3), indicating the expression pattern of a group of genes of the S-39 larvae was as similar as that of CK, whereas significantly different from the S-27. All the nine samples could be clustered into three groups S-27, S-39 and CK based on FPKM of all the 2675 DEGs (Fig. 3b). Heat selection/acclimation induced significant differentiation in gene expression of the rice leaf folder larvae when larvae were exposed to high temperature.

### GO and KEGG analysis of differentially expressed genes

**Fig. 4:**
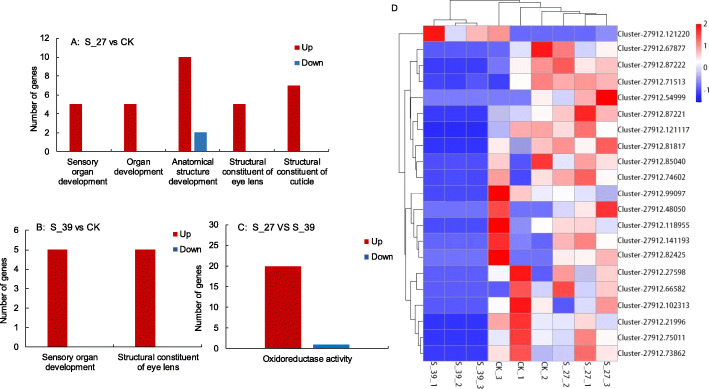
The significant enrichment GO term for DEGs between S-27 and CK (**a**), S-39 and CK (**b**), and S-27 and S-39 (**c**) with the FDR corrected *p*-value of < 0.05. Cluster of nine larvae samples and 21 DEGs enriched in the GO term of oxidoreductase activity based on their expression levels (**d**). Red to blue means the up- to down-regulated level

The unacclimated larvae exposed to 41 °C for 1 h induced 350 DEGs which significantly enriched in these GO terms: sensory organ development, organ development, anatomical structure development, structural constituent of eye lens and cuticle (Fig. 4a). When the heat-acclimated larvae were exposed to 41 °C, they induced 1868 DEGs, and these DEGs were significantly enriched in two GO terms: sensory organ development and structural constituent of eye lens (Fig. 4b). Between the S-27 and S-39, the significantly enriched GO term for 21 DEGs was oxidoreductase activity acting on CH-OH group of donors (Fig. 4c), and these 21 DEGs might be classified as four groups (Fig. 4d). The expression of genes related to oxidoreductase activity was significantly downregulated in the S-39, compared to the S-27, but it was not different between the S-27 and CK (Fig. 4d). Heat acclimation led to downregulation of oxidoreductase activity genes to response to heat exposure.

KEGG pathway enrichment analysis showed that DEGs induced by the heat exposure to 41 °C in both the heat-acclimated and unacclimated larvae were significantly enriched in the same pathways: longevity regulating, protein processing in endoplasmic reticulum, antigen processing and presentation, MAPK signaling, legionellosis, toxoplasmosis,estrogen signaling, and endocytosis, suggesting the general immune or cellular responses to heat stress (Table 1). In the unacclimated larvae, there were eight DEGs enriched in the pathway of spliceosome, whereas in the heat-acclimated larvae there were eight DEGs enriched in the pathway of biosynthesis of unsaturated fatty acids (Table 1). The DEGs between heat-acclimated and unacclimated larvae after heat treatment were signficiantly enriched in metabolism pathways, such as the retinol, porphyrin and chlorophyll, ascorbate and aldarate, and drug metabolism (Table 1). In response to heat stress, the unacclimated larvae mainly upregulated the expression of heat shock protein genes (*HSP70*, *CRYAB*, *HSP27*), *NFYA*, *MEF2C*, *UAP56* and *DHX38*, and downregulated the expression of *SYF1*, *HNRNPUL1*, *PPP5C* and *FGF* genes. Besides these upregulated heat shock protein genes, the heat-acclimated larvae also upregulated other 25 genes and downregulated nine genes including the heat shock protein gene *HSP90A* in response to the heat stress (Table 1).

**Table 1:** KEGG pathway enrichment for the DEGs between S-27, S-39, and CK

KEGG pathway	Log_10_[FDR corrected q-value]			DEGs		
	S-27 vs CK	S-39 vs CK	S-27 vs S-39	S-27 vs CK	S-39 vs CK	S-27 vs S-39
Longevity regulating pathway - multiple species	−20.76	−8.46	ns	**HSPA70, CRYAB**	**HSP70, CRYAB, ADCY5, INSR**, RPS6KB	0
Protein processing in endoplasmic reticulum	−15.08	−6.74	ns	**HSPA70, CRYAB**	**HSP70, CRYAB, HSPBP1, PREB, DNAJC3, BCAP31**, HSP90A, UBE2G2, UBC7, UBE4B	0
Antigen processing and presentation	−12.38	−5.31	ns	**HSPA70, NFYA**	**HSP70, CTSL**, HSP90A	0
MAPK signaling pathway	−10.75	−2.27	ns	**HSP70, HSP27, MEF2C,** PPP5C, FGF	**HSP70, HSP27, MEF2C, PAK1**, SRF	0
Legionellosis	−10.75	−2.96	ns	**HSP70**	**HSP70, CASP7**	0
Toxoplasmosis	−10.40	−2.81	ns	**HSP70**	**HSP70, ITGB1**	0
Measles	−10.34	−2.77	ns	**HSP70**	**HSP70**, GSK3B	0
Epstein-Barr virus infection	−9.20	−2.81	ns	**HSP70, HSP27**	**HSP70, HSP27, EP300, RPB1,** GSK3B	0
Influenza A	−8.12	−1.61	ns	**HSP70, UAP56**, HNRNPUL1	**HSP70, EP300, NS1BP, DNAJC3**, GSK3B	0
Estrogen signaling pathway	−7.81	−2.85	ns	**HSP70**	**HSP70, ADCY5, FKBP4_5,** HSP90A, CALM	0
Spliceosome	−7.39	ns	ns	**HSP70, DHX38, UAP56**, SYF1	0	0
Endocytosis	−5.32	−1.57	ns	**HSP70**	**HSP70, CBL, ASAP, EHD3, CHMP2B, USP8, UBP5, ARFGEF, BIG**	0
Biosynthesis of unsaturated fatty acids	ns	−1.84	ns	0	**HADHA**, *PHS1, ACOX1*, SCD	0
Retinol metabolism	ns	ns	−2.76	0	0	*UGT*, **SDR16C5, RDH12**
Porphyrin and chlorophyll metabolism	ns	ns	−2.57	0	0	*HCCS, UGT*, **hemH**
Ascorbate and aldarate metabolism	ns	ns	−1.65	0	0	**GNL,***UGT*
Drug metabolism - cytochrome P450	ns	ns	−1.65	0	0	*UGT*, GST
Drug metabolism - other enzymes	ns	ns	−1.65	0	0	**UPB1, DPYD**, *UGT*
Metabolism of xenobiotics by cytochrome P450	ns	ns	−1.65	0	0	*UGT*, GST, **EPHX1**
Steroid hormone biosynthesis	ns	ns	−1.60	0	0	*UGT*

ns means q value > 0.05. Bold: the up-regulated gene, normal: the down-regulated gene, italic: either up- or down-regulated genes

The differentially expressed genes between S-27 and S-39 were significantly enriched in eight pathways, seven in which were involved in metabolism and one involved in the steroid hormone biosynthesis. The UDP glucuronosyltransferase family genes (*UGT*) were expressed differentially in all seven pathways between the S-27 and S-29. After expsoure to 41 °C for 1 h, the heat-acclimated larvae downregulated *SDR16C5*, *RDH12*, *hemH*, *GNL*, *UPB1*, *DPYD* and *EPHX1* genes and upregulated *GST* gene, compared to the unacclimated larvae (Table 1).

### Expression levels of oxidoreductase activity-related genes in the heat-acclimated and unacclimated larvae after heat shock

**Fig. 5:**
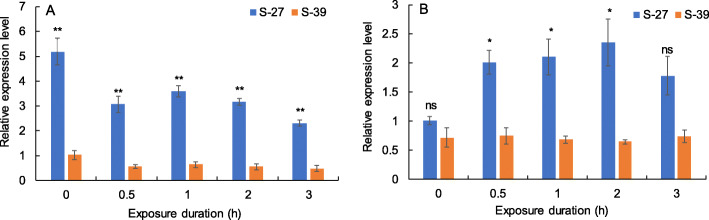
Relative expression levels of two oxidoreductase activity-related genes, putative glucose dehydrogenase *GLD-71513* (**a**) and *GLD-82425* (**b**) in the heat-acclimated larvae (S-39) and unacclimated larvae (S-27) after exposure to 41 °C for 0 to 3 h. ** and * mean significant difference between S-27 and S-39 at the *P* = 0.01/5 and 0.05/5 level, respectively, and ns means no significant difference using the student’s t-test. The error bar represents the standard error (SE)

The expression levels of the oxidoreductase activity-related gene, glucose dehydrogenase (*GLD-71513*) were significantly affected by the heat acclimation (*F*_1, 26_ = 122.025, *P* < 0.001) and the heat exposure duration to 41 °C (*F*_1, 26_ = 13.907, *P* = 0.001, Fig. 5a). The expression levels of another oxidoreductase activity-related gene (*GLD-82425*) were also significantly affected by heat acclimation (*F*_1, 26_ = 10.945, *P* = 0.003), but not affected by the exposure durations to 41 °C (*F*_1, 26_ = 2.042, *P* = 0.165, Fig. 5b). The relative expression levels of oxidoreductase activity-related genes were lower in the heat-acclimated larvae than that in the unacclimated larvae (Fig. 5).

## Discussion

Temperature plays important roles in determining insect survival, development, reproduction and resistance [27, 37, 38]. Insects exhibit obviously physiological and behavioural responses to thermal stress [15, 16, 33, 36]. Moreover, in this study, we found that the rice leaf folder larvae also changed gene expression to respond to heat stress and heat acclimation/adaptation. Under heat stress, the differentially expressed genes of the heat-acclimated and unacclimated larvae were significantly different. The heat-acclimated larvae triggered more genes to respond to heat stress than the unacclimated larvae via changes in gene expression. All larval samples from the S-39, S-27, and CK were distinguished according to the gene expression levels. In the silkworm larvae, the thermotolerant strain induced more DEGs to encounter high temperature than the thermosensitive strain [39]. Insects regulate gene expression levels to respond to heat shock, and the heat acclimated or conditioned larvae trigger more genes involving in this response.

The increased expression levels of heat shock protein genes have been found in insects when exposed to heat stress [20, 21, 36, 40]. Heat shock proteins, such as *HSP40* and *HSP70*, protect insects from direct heat injuries [41, 42]. In this study, we found that both the heat-acclimated and unacclimated larvae increaesd the expression levels of *HSP27*, *CRYAB* and *HSP70*, when encountering the heat exposure to 41 °C, but addintionally, the heat-acclimated larvae decreased the expression of the *HSP90A*. The global transcriptome results indicated that *HSP* genes might have different expession patterns in the heat-acclimated and unacclimated larvae. *HSP70*, *HSP27*, and *CRYAB* genes were upregulated in both the heat-acclimated and unacclimated larvae exposed to high temperature, suggesting that these genes might be involved in the rapid response to heat stress, but the *HSP90A* was downregulated or did not change in the heat-acclimated larvae when exposed to heat, which might be involved in the slowly developing heat acclimation or adaptation. The RT-qPCR detection also supported this expression mode of *HSP70* and *HSP90* in the rice leaf folder larvae [36]. Heat shock protein gene family are involved in the response and acclimation to heat stress.

Sensory organ plays important roles in responding to and tolerating heat stress. In the present study, when larvae were exposed to 41 °C for 1 h, the significantly enriched GO terms for DEGs in the unacclimated larvae were involved in the sensory organ development, structural consitituent of eye lens, and structural consitituent of cuticle, but the enriched GO terms in the heat-acclimated larvae were involved in the sensory organ development and structural consitutent of eye lens, but not the cuticle. Sensory organs of larvae, such as eyes and cuticle are sensitive to heat stress and may sense this stress. Therefore, the sensation-related genes may play important roles in rapid response to heat stress. Heat induces changes in cuticle, such as cuticular hydrocarbon profile [25, 43, 44]. After multigenerational heat acclimation, the sensitivity of cuticle to heat may become lower than before, and therefore, the heat-induced DEGs are not enriched in the GO term of structural consitituent of cuticle anymore. Cuticle protein genes are involved in the cuticle formation, and they are necessary for cuticle development, flexibility, and metamorphosis [45]. Moreover, expression of the cuticle protein genes is also related with the survival of insects [45, 46]. Transcriptional patterns of genes encoding cuticle proteins in the water flea *Daphnia pulex* have responses to the interaction between biotic (predator presence) and abiotic (low calcium concentration) environmental stresses [47]. In rice planthoppers, low temperature induces the differential expression of four cuticle-related genes, but these genes are not induced by a high temperature [40]. In this study, we found that cuticle protein genes in the rice leaf folder larvae were expressed differentially induced by heat exposure. Cuticle protein genes may be involved in the response of this insect to heat stress. When larvae have acclimated to a high temperature, they do not regulate expression of genes related to structural consitituent of cuticle to response to heat stress, but they still regulate expression of genes related to structural consitituent of eye lens. Therefore, we presumed that the cuticle could acclimate to heat but not the eye lens.

We found that both the heat-acclimated and unacclimated larvae were significantly enriched for DEGs in similar KEGG pathways related to longevity regulating, protein processing in endoplasmic reticulum, immunity, MAPK and estrogen signaling, and diseases. These pathways might be involved in the response of the rice leaf folder larvae to heat stress. It has beeen found that the protein turnover, immune processes and signal transduction pathways had a close relationship with heat tolerance of *Glyphodes pyloalis* larvae [48]; several immune-related genes were downregulated when the fifth instar larvae of silkworm exposed to 37 °C for 9 h [20]. In this study, furthermore, we found that the different KEGG pathways enriched for DEGs in the heat-acclimated and unacclimated larvae when they were exposed to heat stress. For example, the pathway of spliceosome was enriched for DEGs in the unacclimated larvae, showing that *DHX38* and *UAP56* genes were upregulated and *SYF1* gene dowregulated, but not enriched in the heat-acclimated larvae. On the contrary, pathway of the biosynthesis of unsaturated fatty acids was significantly enriched for DEGs in the heat-acclimated larvae, but not in the unacclimated larvae. Therefore, we speculated that splicing of mRNA and biosynthesis of unsaturated fatty acids might play roles in heat acclimation or resistance of insects.

The DEGs between the heat-acclimated and unacclimated larvae were mainly enriched in the GO term of the molecular function on oxidoreductase activity when larvae were treated by high temperature. In rice planthoppers, oxidoreductase enzyme genes were also expressed differentially under high temperature [40]. These results show that oxidoreductases play important roles in the developmental or physiological process, such as ecdysteroid and glucose metabolism. RNA inference of ecdysone oxidase results in the accumulation of ecdysteroid and death of larvae or pupae of silkworms [49]. The expression of glucose-6-phosphate dehydrogenase (G6PD) is correlated with the longevity of *D. melanogaster*, and the mean life spans of G6PD over-expressed flies are extended [50]. The metabolic pathways play a significantly role in the high temperature tolerance of silkworms [39, 51]. In the rice leaf folder, we found that the significant differently expressed genes between heat-acclimated and unacclimated larvae were mainly involved in metabolism of retinol, porphyurin and chlorophyll, ascorbate and aldarate, and drug. Genes *UGT*, *RDH*, *SDR* and *GST* were expressed differentially between heat-acclimated and unacclimated larvae in response to heat stress. Uridine diphosphate (UDP)-glucuronosyl transferase (UGT) is a family of enzymes that catalyzed the glucuronidation [52]. UGTs play important roles in detoxification [53], olfaction [54], UV-shielding [55], and pigmentation [56] in insects. We found that after treatment at 41 °C for 1 h, expression of *GSTs* was significantly up-regulated in the heat-acclimated larvae, compared to the unacclimated larvae. Glutathione S-transferase (GST) is a family of detoxification enzymes in herbivorous insects [57]. The upregulation of oxidoreductase activity may effectively remove the accumulation of toxic substances induced by heat stress, and consequently improve the ability of larvae to tolerate the higher temerpature and slowly develop heat acclimation.

**Fig. 6:**
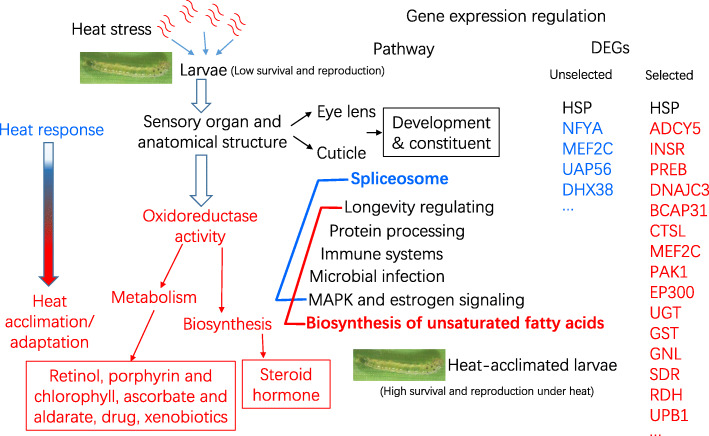
A presumptive mode for the gene-mediated rapid response and slowly developing acclimation of insect larvae to heat. The red parts mean items only belonging to the heat-acclimated larvae, the blue parts belonging to the unacclomated larvae, and black parts belonging to both the heat-acclimated and unacclimated larvae

Based on these results, a presumptive two-stage molecular regulation mode of insect larvae to cope with heat stress has been established in this study (Fig. 6). The first stage is the rapid response of insects to heat. These genes related to sensory organ development and structural constituent of eye lens and cuticle are involved in the response. In this stage, heat shock protein genes play important roles in longevity regulating, protein processing in endoplasmic reticulum, antigen processing and presentation, MAPK signaling, estrogen signaling, and spliceosome pathways, showing a significantly upregulation to response to heat stress. Insects surviving under heat stress may furtherly induce the second stage of regulation, acclimation or adaptation. In this stage, insect cuticles can tolerate heat stress, and expression of structural constituent of cuticle-related genes is not affected by heat. The oxidoreductase activity-related genes are down-regulated to alleviate the heat injuries. Moreover, the insects change expression levels of genes related to biosynthesis and metabolism, such as biosynthesis of unsaturated fatty acids and steroid hormone, metabolism of retinal, porphyrin, chlorophyll and drug. These changes improve heat tolerance of insects and develop heat acclimation (Fig. 6).

## Conclusions

The larvae of rice leaf folder acclimated to heat stress via multigenerational selection. The heat-acclimated larvae regulated expression of more genes to response to heat shock than the unacclimated larvae. Genes related to sensory organ development and structural constituent might modulate the rapid response of larvae to heat stress, and genes involved in the oxidoreductase activity might be associated with heat acclimation. The sensation, biosynthesis and metabolism-related genes play roles in rapid response and slowly developing acclimation of insects to high temperature.

## Methods

### Insects

The experimental population of rice leaf folders was collected from a rice field in Nanjing, an experimental station of Nanjing Agricultural University at Jiangpu (longitude 118.62^o^ E, latitude 32.02^o^ N), Jiangsu province, China, and then reared in climate chambers using corn seedlings for 3 years [58], and then reared using wheat seedlings for 2 years at 27 ± 1 °C, (60 ± 5) % RH and a 14 L: 10D photoperiod [59].

### Heat selection

Heat treatment was executed for the third-instar larvae in climate chambers by reference to Gu et al. [36]. The temperature in the chamber increased or decreased by 3 °C in 8 min. The temperature in chamber was set at 27, 30, 33, 36, and 39 °C at 9:00, 9:30, 10:00, 10:30, and 11:00 am, respectively. After 3 h, the temperature was set at 36, 33, 30, and 27 °C at 14:00, 14:30, 15:00, and 15:30 (local time), respectively, and maintained at 27 °C until the heat treatment was performed again in the next day. After three successive days of this heat treatment, the survival rate of treated larvae was observed, and then these larvae were reared at 27 °C until the next generation of heat treatment. The data of survival rates were analysed using a GLM treating temperature as a fixed effect and generation as a random variable. The differences in survival rates between the heat selected and unselected larvae were analysed using the Mann-Whitney U test, because the data in the sixth generation of heat selection did not meet the normal distribution. The survival rates after heat treatment during 5–9 generations became as similar as the control at 27 °C (Fig. 1), and therefore, we considered the heat treated population as a heat-acclimated strain and the population reared at 27 °C as an unacclimated strain.

### Heat exposure on the heat-acclimated and unacclimated strains

When the heat-acclimated strain was selected for 15 generations at 39 °C, 30 third-instar larvae were collected and transferred onto wheat seedlings. Then these larvae were exposed to 41 °C for 1 h in a chamber with wheat seedlings. Thirty larvae from the unacclimated strain were also performed this heat treatment as the heat-acclimated larvae. After heat exposure, four larvae were collected and put into a 2 ml tube as a sample. All samples were frozen in liquid nitrogen. These larval samples exposed to 41 °C were named S-39 and S-27 for the heat-acclimated and unacclimated larvae, respectively. The third instar larvae collected from the unacclimated strain and reared at 27 °C were control (CK). Three replications were performed for S-27, S-39 and CK. A total of nine larval samples were collected.

### RNA extraction, cDNA library preparation and sequencing

Total RNA of each nine larval samples collected above was isolated using Trizol Reagent (Takara, Dalian, China) followed the manufacturer instructions. The mRNA was concentrated using magnetic beads with Oligo (dT), and then broken into fragments and synthesized double stranded cDNA via inverse transcription. Finally, nine cDNA libraries were constructed. All nine libraries were sequenced on an Illumina Hiseq-2000 platform at the Novogene Experimental Department, Beijing Novogene Bioinformatics Technology Co. Ltd. The read length during the paired-end sequencing was 150 bp. The raw data were submitted to NCBI (NCBI, Bethesda, USA) with the accession number SRR11610902.

### Transcriptome assembly and function annotation

The raw reads containing adapter and ploy-N, or with low-quality bases were removed and then the clean reads were obtained. The Q20, Q30, GC-content and sequence duplication level of these clean reads were analysed, and the results were showed in Table S1. Transcriptome assembly was performed based on clean reads using Trinity [60]. The sequences were aligned with following databases for homology annotation: NCBI non-redundant protein (Nr) and nucleotide sequences (Nt), Protein family (Pfam), Clusters of Orthologous Groups of proteins (KOG/COG), Swiss-Prot (a manually annotated and reviewed protein sequence database), KEGG Orthology (KO), and Gene Ontology (GO). The completeness of the transcriptome was analysed using BUSCO version 3.0.2 [61]

### Quantification of gene expression levels

Gene expression levels were established using RSEM for each sample [62]. Differential expression analyses of genes between two samples were performed using the DESeq R package for FPKM data with biological replicates. The *P* values were corrected using the Benjamini and Hochberg’s method to control the false discovery rate (FDR). Genes with a fold-change> 2 and a FDR corrected *p*-value of < 0.05 found by DESeq were considered as differentially expressed genes (DEGs).

### GO and KEGG pathway enrichment analysis

In order to adjust the gene length bias in DEGs, GO enrichment analysis of DEGs was performed using the GOseq R packages based on Wallenius non-central hyper-geometric distribution [63]. The enrichment of DEGs in KEGG pathways was analysed using the KOBAS software [64]. The GO term and KEGG pathway with a FDR corrected *p*-value of < 0.05 were considered as enrichment in DEGs.

### Clustering analysis on samples based on DEGs

In order to explore the differences in gene expression between S-27, S-39 and CK, the clustering for DEGs and nine samples was analysed using the hierarchical clustering method in R software based on the FPKM values. Principal component analysis (PCA) based on FPKM of all DEGs was also performed to distinguish these nine samples via analyzing similarities in two major components PC1 and PC2 [65].

### Expression levels of genes related to oxidoreductase activity under heat stress

The 3rd-instar larvae collected from the heat-acclimated (S-39) and unacclimated (S-27) strains were exposed to 41 °C for 0, 0.5, 1, 2, and 3 h. The total RNA was extracted using Trizol method (Takara, Dalian, China) from five third-instar larvae after heat exposure to measure the expression levels of two putative glucose dehydrogenase (GLD) genes (*GLD*-*71513* and *GLD*-*82425*) related to oxidoreductase activity, using the RT-qPCR method. The DNA sequences of these two genes were listed in Table S4. 1000 ng RNA of each sample was used for synthesizing the single-stranded cDNA using PrimeScript RT Reagent Kit with gDNA Eraser (Takara, Dalian, China). *β-Actin* and *PRs15* genes were used as internal reference genes [66, 67]. Gene-specific primers were designed using Primer Premier software (version 5.0) and Primer-BLAST on NCBI (Table S5), and the primer specificity was verified via the dissociation curve analysis and agarose gel electrophoresis. The amplification efficiencies of all primers were 95.6–99.5% (Table S5). The RT-qPCR was performed using TB GREEN Premix Ex Taq Kit (Takara, Dalian, China) in an ABI 7500 (Applied Biosystems, Carlsbad, CA, USA). The reaction mixture was 20 μL, containing 10 μL TB GREEN Premix Ex Taq, 0.4 μL of 10 μM forward and reverse primers (Table S5), 0.4 μL ROX reference Dye II, 2 μL cDNA and 6.8 μL ddH_2_O. The cycling parameters were 95 °C for 30 s, followed by 40 cycles of 95 °C for 5 s, 60 °C for 34 s, followed by melting curve analysis to determine the specificity of PCR products. The cycle threshold (Ct) was normalized by the reference gene (ΔCt). The 2^-ΔΔCt^ method was used to calculate the relative expression level of a gene in a sample [68]. The geometric mean of two relative expression levels based on two reference genes was considered as the expression level of a target gene. Each sample was measured three times, and three biological replications were performed for each heat exposure. The relative expression levels of genes between S-39 and S-27 treated at 41 °C for different exposure durations were analysed using a GLMM, and the student’s *t* test was used to differentiate relative expression levels between S-39 and S-27 followed by the Bonferroni correction (*P* < 0.01/5 or 0.05/5), because all data met the normal distribution.

## Supplementary information

**Additional file 1: Table S1.** Quality of RNA-seq data. **Table S2.** Statistics of the sequencing and assembly data (bp). **Table S3.** Statistics of unigene annotation. **Table S4.** Sequences of the two putative glucose dehydrogenase genes (*GLD*) in the transcriptome. **Table S5.** Primers for RT-qPCR in this study. **Figure S1.** Length distribution of unigene and transcript. **Figure S2** The BUSCO analysis of the transcriptome.

## Data Availability

All data analyzed during this study are provided in this published article and additional files. The sequence data of this study have been deposited into Sequence Read Archive (SRA), accession number SRR11610902. (https://www.ncbi.nlm.nih.gov/sra/SRR11610902).

## Abbreviations

COG: Clusters of Orthologous Groups of proteins database
Ct: Cycle threshold
DEGs: Differential expressed genes
FPKM: Fragments per kilobase of transcript sequence per millions base pairs sequenced
GLD: Glucose dehydrogenase
GLM: General linear model
GLMM: Generalized linear mixed model
Nr: NCBI nonredundant database
Nt: NCBI Nucleotide sequence database
Pfam: Protein families database
RT-qPCR: Quantitative real-time PCR
