# Supplementary material for: Comparative transcriptome analysis of the rice leaf folder (Cnaphalocrocis medinalis) to heat acclimation
Source: BMC Genomics. 2020 Jun 30;21:450. doi: 10.1186/s12864-020-06867-6 (PMC7325166; doi:10.1186/s12864-020-06867-6)
Supplement: Supplementary file 1 — Additional file 1: Table S1. Quality of RNA-seq data. Table S2. Statistics of the sequencing and assembly data (bp). Table S3. Statistics of unigene annotation. Table S4. Sequences of the two putative glucose dehydrogenase genes (GLD) in the transcriptome. Table S5. Primers for RT-qPCR in this study. Figure S1. Length distribution of unigene and transcript. Figure S2 The BUSCO analysis of the transcriptome. [file 12864_2020_6867_MOESM1_ESM.docx]

**Comparative transcriptome analysis of the rice leaf folder (*Cnaphalocrocis medinalis*) to heat acclimation**

Peng-Qi Quan, Ming-Zhu Li, Gao-Rong Wang, Ling-Ling Gu, Xiang-Dong Liu*

Department of Entomology, Nanjing Agricultural University, Nanjing 210095, China

*Corresponding author: liuxd@njau.edu.cn

**Table S1** Quality of RNA-seq data.

| Sample | Raw reads | Clean reads | Clean bases (G) | Error (%) | Q20 (%) | Q30 (%) | GC content (%) |
| --- | --- | --- | --- | --- | --- | --- | --- |
| CK-1 | 51,831,776 | 50,680,722 | 7.60 | 0.03 | 95.88 | 93.67 | 50.27 |
| CK-2 | 51,000,628 | 49,749,850 | 7.46 | 0.03 | 96.96 | 95.12 | 50.28 |
| CK-3 | 47,631,668 | 46,469,076 | 6.97 | 0.02 | 97.85 | 96.55 | 50.31 |
| S-27-1 | 50,933,024 | 49,405,998 | 7.41 | 0.02 | 97.91 | 96.63 | 52.34 |
| S-27-2 | 45,545,452 | 44,520,666 | 6.68 | 0.03 | 97.02 | 95.24 | 51.94 |
| S-27-3 | 63,146,340 | 61,485,908 | 9.22 | 0.03 | 96.85 | 94.95 | 53.48 |
| S-39-1 | 63,611,900 | 62,371,284 | 9.36 | 0.03 | 97.04 | 95.25 | 51.96 |
| S-39-2 | 58,296,806 | 62,371,284 | 8.59 | 0.03 | 96.73 | 94.78 | 50.38 |
| S-39-3 | 57,396,390 | 56,307,794 | 8.45 | 0.03 | 97.05 | 95.27 | 52.81 |

**Table S2** Statistics of the sequencing and assembly data (bp).

|  | Min Length | Mean Length | Median Length | Max Length | N50 | N90 | Total Nucleotides |
| --- | --- | --- | --- | --- | --- | --- | --- |
| Transcript | 201 | 791 | 420 | 34,035 | 1,344 | 298 | 228,684,988 |
| Unigene | 201 | 1,040 | 660 | 34,035 | 1,583 | 451 | 199,737,840 |

**Table S3** Statistics of unigene annotation.

|  | Number of unigene | Percentage (%) |
| --- | --- | --- |
| Annotated in NR | 79,541 | 41.43 |
| Annotated in NT | 45,144 | 23.51 |
| Annotated in KO | 26,784 | 13.95 |
| Annotated in SwissProt | 51,070 | 26.6 |
| Annotated in PFAM | 59,204 | 30.83 |
| Annotated in GO | 59,675 | 31.08 |
| Annotated in KOG | 28,940 | 15.07 |
| Annotated in all Databases | 9,609 | 5 |
| Annotated in at least one Database | 103,535 | 53.93 |
| Total Unigenes | 191,974 | 100 |

**Table S4** Sequences of the two putative glucose dehydrogenase (GLD) genes in the transcriptome

| Gene Id | Sequence |
| --- | --- |
| *GLD*-71513  [Cluster-27912.71513] | TTTGGCTACGGGACCTTAAAAAGTGAAAAACTTTTACCAGTAACATCCGTCATGACGTCAACAGTGGAAGCTGTAGTGAGCTCTGTACGGGCCATACAGGGTGCCTTACTAGTGATTGCAGGGCTACAGCTGACAGGGTACATGTATCCACAGTCCACTACCGTTACAAATGGAGCCTCATATGATTTTATTATATGCGGACCGGCACTGGAGGGAGTGTCATTGCGAACAGACTGACAGAAATCCCCAACATTAATGTACTCCTTATTGAAGCTGGGGATGATCCTAGAATAGAATCATTACTTCCAGGTTTGCTGACGCTACAGCCTCTCACGGAACAGGACTTCAACTTCACTTCTGAAGACGATGGTAGAACAGGACAGTACCTCAAAGACAGGGTAGTGGGTATGTCTCAAGGGAAAATGCTGGGAGGTAGCAGTAGCTTGAACCATCTCATTCATGCAAGAGGAAACGCTAGGGACTACCAAAGGTGGGCAGACGCAGCCCAAGACGAATCCTGGAACTTTGACAATCTCATGCAGTATTTCATTAAGAGTGAGAAAGTTGAGGATGAAGACATCCTCAAATCTAAACTTGGGAAATACCACGGAACAGAAGGCTATTTAAGAGTATCTACTCAACGCAGTGACGAGAACATACCAATTTTTGAGGCTTTCGCTGAATTAGACCACGAGAATGTTTTAACAGCTAACACTCCAGAATTTACAGTCGGTATTACAGAACCTTTGCTGAACATTGCTGATGGCAAAAGACAAAGTACTGCAGAATATTTGAGACCACTCTCGGACCGTCCTAATTTCCATATAATGAAAAATACACACGTGACTAAAGTATTGTTTGATGATGACAGGAATGCTATTGGCGTGGAAGCTGTTACAGCTGGTGGAGAAACTGTTACGATCAACGCAAATGTTGAGGTTATTTTAGCAGCCGGTGCTTTTGTGACTCCTCAACTACTTATGCTTTCCGGAGTTGGCCCCAAAGATCATTTAGAGTCCTTCAATATTGATGTTATCTCAGATTTGCCTGTTGGCAAGAACCTCCAAGATCACGTAGATCCAGTAGTTATCCATGCGTTACAAGAAGGTGAAGCTCCTACTGCTCCAGCCAATCCTCACGAATATCCAGTACCAACTACCGTTGCTTACACTGCTCTAGACATGAATCAAGGGTATCCTGACTACCAAACGATAAACTTGCTCTTCCCTCCAGACTCCACAGGTTTGATTCAGTTCTGCTCTTTAGTCTTTGCTTATAACGACGAGATTTGTCAAAAATGGTTTGATGGTGGTAAAGGGCGGTATACTTTATTCACCTGCCATAATCTTATGCAGCCGTATTCAACTGGAGAAGTTCTCCTCCGTAGTGCAGACCCGAAAGACCCTCCTATCATCCACCACGGAATCTTCTCAAACGAAACTGATCTACACAACATGGCGTTGTACCTTAAAGACTTCGCTCGCTTCGGCAAAACGTCGTATATGAAAAATGTTGGAGGTTTCTTGATTGATCTGGAATTGGAAGAATGCAAAGGTTTATCAAAGGATTCTTATGAATACTGGAGATGTTACGCCCTGAGTATGTCGGCTACTTTATGGCACTACAGCAGCACAGCGTCTATGGGACCGGTTCTGGACAGCCACTTGAGAGTTAAAGGAGTGAACAGGCTGAGGGTTGCCGATTCTAGTGCCATGCCTAATACCGTAAGTGGTAACCCAATGGCAGCGGTGGTGGCGCTTGCAGAAAAAGCAGCAGATTTGATTAAGAAAGATAATGGTGCAAGTCCCAGAAGTAGCAACTAA |
| *GLD*-82425  [Cluster-27912.82425] | GTGTCTACTCTTCAAGCAGGAGAACCCGTAACGACACCAGCTAACCCTCATTTGTTCCCCGTCCCCATCACAACAGGGTACCATGCCCTTGACAAGTCGCAGTCATACCCTGACTACCAGACTATTAATCTGCTATTCCCTGCCGATAACACAGCTCAGATTCAAATCACATCTATGGTATTTGGCTTCAACAACAACATCGCTCAGCAATTCTTCGATGGTGGTGCTGGACGCGCCACTCTGATCTCTATCCCCAACATCATGCAGTCTCTTTCCCGAGGGGAAGTCCTTCTGAACAGCACAGATTACAAAGACGATCCTATCATCCGCCACGGCATTTACTCGAATGAAGCTGACCTCCACAATATGGTTTTGTACTTGCAAGACTTTTCTCGCATTTACAACACCACCGCATTCAAGTCTATAGGAGCAAAAATGATTGACTTGGATCTGCCTGCATGCAATAATATCAAGAGGGGCACGTATGAATATTGGAGGTGTTACGCTATAGAAACGTCTGCATCTTTGTGGGCGTACACCAGCACAGCGAGGATGGGAACTGTTCTGGACAGCCGTCTGCGCGTACGAGGAGTCCACGGGGTTCGTGTGGTAGACGCCAGTGCTATGCAGAACTCCATCAGTGGAAACATCCATGCTGGTGTGTTTGGTCTTGCTGAGAGAGCGGCTGACCTGGTCAAGGCTGACTGGGGAGCGTCGCCCAGAGGCATGGAATAG |

**Table S5** Primers for qPCR in this study

| Gene | Primer Sequence（5’-3’） | Size (bp) | Amplification efficiency |
| --- | --- | --- | --- |
| *β-Actin*-sense | ATGGTCGGCATGGGACAG | 153 | 98.58% |
| *β-Actin*-anti | GAGTTCATTGTAGAAGGTGT |  |  |
| *RPs15*-sense | ACGTACCCGCTTACAAACCC | 156 | 96.40% |
| *RPs15*-anti | TGACCAAGGTGAGCAACAGAG |  |  |
| *GLD-82425*-sense | ATTTGTTCCCCGTCCCCATC | 109 | 95.63% |
| *GLD-82425*-Anti | TGAGCTGTGTTATCGGCAGG |  |  |
| *GLD-71513*-sense | TATGCAGCCGTATTCAACTGG | 137 | 99.46% |
| *GLD-71513*-anti | AGCGAGCGAAGTCTTTAAGGTA |  |  |

**Fig. S1** Length distribution of unigene and transcript.


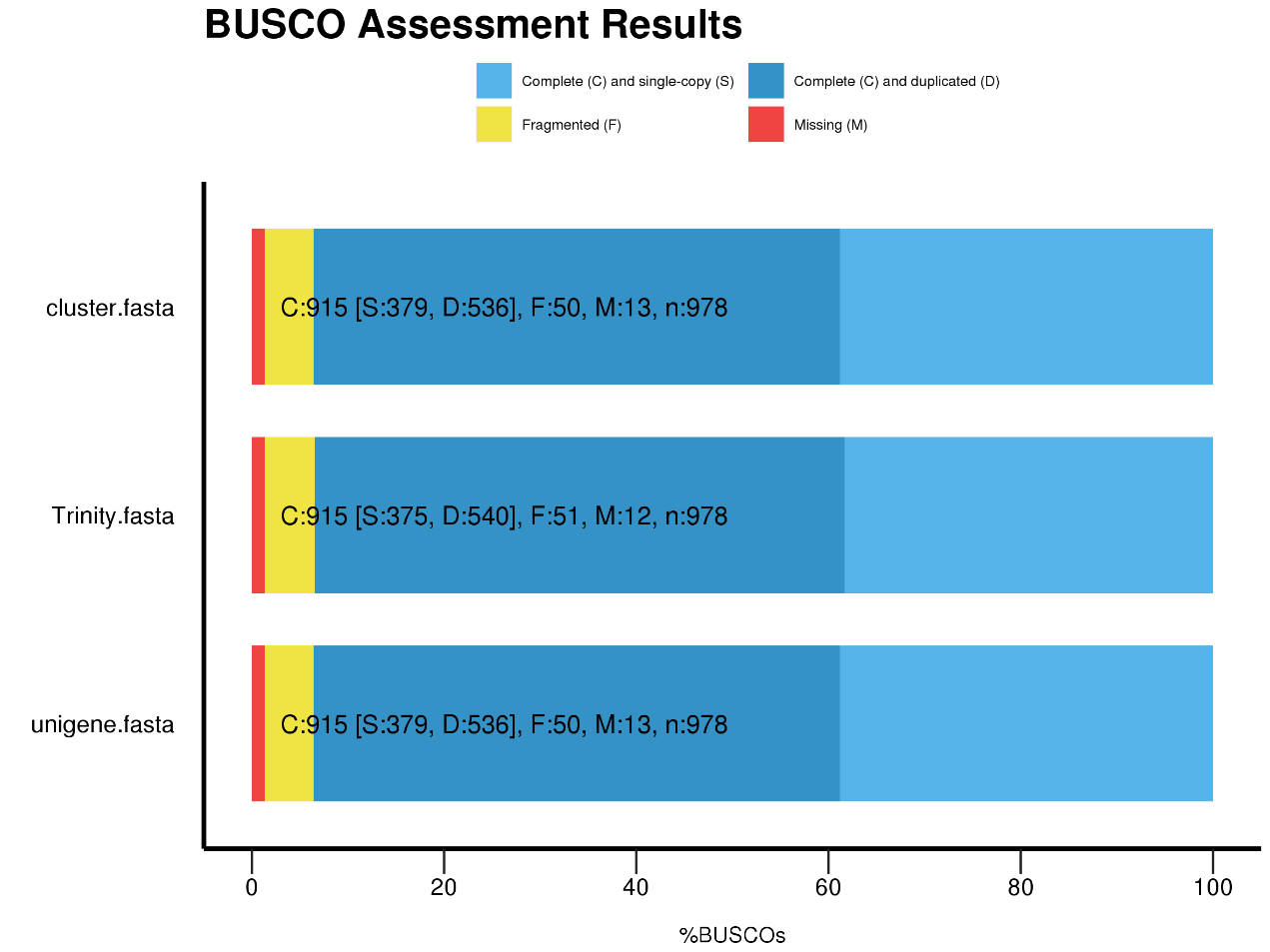


**Fig. S2** The BUSCO analysis of the transcriptome
